# Intra-articular hip injections for lateral hip pain

**DOI:** 10.1093/jhps/hnu012

**Published:** 2014-10-08

**Authors:** Matthew C. Bessette, Joshua R. Olsen, Tobias R. Mann, Brian D. Giordano

**Affiliations:** Department of Orthopaedics and Rehabilitation, University of Rochester Medical Center, 601 Elmwood Ave, Box 665, Rochester, NY 14642, USA

## Abstract

Occult intra-articular hip pathology is commonly found in patients with greater trochanteric pain syndrome, and may be a possible pain generator in patients with recalcitrant lateral hip pain. We investigated the effect of intra-articular hip injections in patients with recalcitrant lateral hip pain. Between September 2012 and May 2013, patients over the age of 18 with a history lateral hip pain who had received prior treatment with non-steroidal anti-inflammatory medications, physical therapy and peritrochanteric corticostroid injections were enrolled. Treatment consisted of an ultrasound guided intra-articular corticosteroid injection followed by a course of directed physical therapy and a non-steroidal anti-inflammatory medication. Patients performed GaitRite analysis at baseline and 12 weeks following the injection. In addition, the Modified Harris Hip Score (mHHS), Non-Arthritic Hip Score (NAHS), Hip Outcome Scores (HOS), Short Form 36 (SF-36) and a visual analogue pain score (VAS) were collected at baseline, 1, 6 and 12 weeks.A total of 16 patients were studied. Patients experienced significant improvements from their baseline mHHS at 1 and 12 weeks (*P* = 0.03, *P* = 0.04). The minimal clinically important difference (MCID) was exceeded at multiple timepoints on various clinical outcome surveys. Velocity and stride length were not significantly improved at 12 weeks. Intra-articular hip injections may decrease pain and improve function in patients with recalcitrant lateral hip pain, and occult intra-articular hip pathology should be considered in the etiology of lateral hip pain. Though low enrollment numbers left this study underpowered, MCID comparisons demonstrated potential benefit from this treatment.

## INTRODUCTION

Greater trochanteric pain syndrome (GTPS) was first coined in 1958 and is now used to describe the common, yet not fully understood condition of lateral hip pain. It is characterized by tenderness to palpation over the greater trochanter and can encompass a number of disease processes [1]. It is thought to affect between 10 and 20% of the general population, and to be more prevalent in certain populations, such as those reporting low back pain (LBP), the middle-aged and elderly and women [2]. It has been shown to confer levels of disability and quality of life similar to those associated with end-stage hip osteoarthritis (OA) [3].

Treatment of this condition is typically amenable to non-operative modalities such as physical therapy (PT), non-steroidal anti-inflammatory medications (NSAIDs) and local corticosteroid injections [4]. When these fail to provide satisfactory relief from symptoms, it is postulated that occult intra-articular pathology of the ipsilateral femoroacetabular joint may be a driving force in the causation of lateral hip pain. By altering local and more global body mechanics, intra-articular pathology could provoke alterations that change forces affecting lateral hip structures. Articular hip pain typically causes anterior or medial hip pain, but resultant global hip dysfunction could manifest as more lateral or posteriorly based hip pain. Our hypothesis is that an injection of corticosteroid into the femoroacetabular joint may provide significant relief of GTPS symptoms in cases refractory to other non-surgical efforts. Furthermore, intra-articular injections may provide useful diagnostic information regarding the primary etiology of pain generation for a particular hip condition.

## MATERIALS AND METHODS

Between September 2012 to May 2013, patients over the age of 18 with a history of GTPS were enrolled from an academic clinic setting to receive an ultrasound guided intra-articular hip injection by a single sports medicine trained orthopedic surgeon specializing in hip preservation surgery. The diagnosis of GTPS was made clinically, although some patients had magnetic resonance imaging (MRI) available at the time of initial consultation. Inclusion criteria required patients to have lateral hip pain for at least 6 months of a non-traumatic etiology, at least one prior trochanteric bursa corticosteroid injection and at least one course of NSAIDs and PT. Table I summarizes the pre-treatment clinical and injection history as well as available imaging studies. Patients were excluded if they had obvious evidence of spine pathology, radiographic hip OA (joint space less than 2 mm), bilateral symptoms, previous surgery or trauma to the hip or pelvis, a history of dysplasia or other developmental abnormality or a trochanteric steroid injection within the past 3 months.

**Table I.** Pre-injection participant treatment and radiographic findings

Subject	Number of previous peritrochanteric injections	Duration of lateral hip pain symptoms (months)	Physician summary of MRI findings	Joint space width on standing AP pelvis radiograph (mm)
1	2	66	Labral tear and abductor tendinopathy	3.6
2	3	78	Abductor tendinopathy and mild DJD	3.9
3	3	18	Partial thickness abductor tear and mild DJD	No radiographs available
4	2	12	Abductor tendinopathy, trochanteric bursal inflammation and labral tear	4
5	1	6	Partial abductor tearing and bursal inflammation	2.7
6	2	24	No MRI available	4.2
7	2	6	Abductor tendinopathy and labral tear	4.7
8	1	6	Labral tear	5.1
9	3	10	Labral tear, paralabral cyst and moderate DJD	2.9
10	1	60	No MRI available	4.3
11	1	12	Partial-thickness gluteus medius tearing	5.7
12	1	6	Labral tear	5.8
13	10	78	Partial-thickness abductor tearing and mild DJD	3.5
14	1	12	Labral tear, abductor tendinopathy and mild DJD	3.5
15	1	6	Labral tear	5.1
16	2	12	Partial abductor tearing and mild DJD	3.5

DJD, degenerative joint disease.

Treatment consisted of an ultrasound-guided corticosteroid injection (2 cm^3^ of 6 mg/cm^3^ Celestone and 4 cm^3^ of 0.25% Marcaine without epinephrine) preformed in clinic by the senior author (B.G.) using 2 cm^3^ of 1% Lidocaine without epinephrine for local anesthesia. This was followed by a course of directed PT and NSAIDs. PT consisted of a functional hip protocol conducted by a therapist familiar with hip-specific exercises. Meloxicam 7.5 mg was prescribed to be taken twice daily for a total course greater than 30 days if tolerated.

Patients performed GaitRite (CIR Systems, Inc, Sparta, NJ, USA) analysis at baseline and 12 weeks following their intra-articular injection. In addition, a Modified Harris Hip Score (mHHS), Non-Arthritic Hip Score (NAHS), Hip Outcome Scores (HOS), Short Form 36 (SF-36) and a visual analogue pain score (VAS) were collected at baseline, 1, 6 and 12 weeks after injection. Radiographs were examined for minimum joint space width [5]. Patients’ electronic records were reviewed for their clinical history.

All data were collected and stored in Microsoft Excel (Microsoft, Redmond, WA, USA). A two-tailed student’s *t*-test assuming equal variance was used to compare outcome scores. *P* values of less than 0.05 were considered statistically significant.

The minimal clinically important difference (MCID) for the various patient-reported outcome surveys were estimated using similar existing studies if available. For the mHHS, the MCID was considered to be an 8% change from baseline [6, 7] while changes of nine and six were used for the HOS ADL and Sports subscales [8]. A 12% change from baseline was used for the SF-36 [9]. A reasonable approximation for the NAHS was not identified.

## RESULTS

A total of 16 patients consented to take part in the study after being identified in clinic and deemed eligible by inclusion and exclusion criteria. All patients were female with an average age of 57.1 ± 6.7 years, an average height of 165.1 ± 4.4 cm, an average weight of 75.5 ± 15.4 kg and an average BMI of 28.2 ± 4.2 kg/m^2^. No adverse events were reported in relation to the study. Patients received an average of 2.3 ± 2.2 peri-trochanteric injections prior to receiving an intra-articular injection. Their symptoms had been present for 25.8 ± 27.4 months, and their pre-injection standing AP pelvis radiographs demonstrated an average of 4.2 ± 0.9 mm of joint space.

Data from mHHS, NAHS, HOS and SF-36 surveys as well as a VAS were collected from all 16 patients at baseline, 9 (56%) at 1 week, 10 (63%) at 6 weeks and 7 (44%) at 12 weeks. The results are summarized in Table II.

**Table II.** Outcome scores

	Baseline	1 week	6 weeks	12 weeks
HOS				
ADL	65.0 ± 12.9	74.4 ± 12.6^b^	73.2 ± 16.1^b^	71.9 ± 23.8^b^
Sports	51.3 ± 18.7	58.6 ± 21.2^b^	63.36 ± 23.0 ^b^	55.1 ± 24.9
NAHS	61.4 ± 10.5	69.8 ± 14.4	69.4 ± 12.1	68.1 ± 15.0
mHHS	56.2 ± 10.7	65.9 ± 7.7^a^^,^^b^	64.2 ± 13.5^b^	69.0 ± 18.9^a^^,^^b^
SF-36				
Physical function	46.3 ± 15.1	59.4 ± 23.8^b^	51.0 ± 17.3	57.1 ± 26.1^b^
Role physical	27.1 ± 35.9	38.9 ± 48.6^b^	23.3 ± 38.4	51.2 ± 47.2^b^
Bodily pain	52.0 ± 15.2	65.3 ± 16.9^b^	53.8 ± 14.3	64.3 ± 15.2^b^
General health	71.5 ± 18.7	66.1 ± 18.5	74.0 ± 19.0	77.9 ± 16.5
Vitality	62.8 ± 23.2	63.3 ± 24.7	57.0 ± 30.0	74.3 ± 13.0^b^
Social function	69.5 ± 15.8	80.6 ± 9.1^a^	53.7 ± 14.3	68.8 ± 15.3
Role emotional	68.7 ± 39.4	81.5 ± 33.8^b^	53.3 ± 42.2	66.7 ± 47.1
Mental health	81.0 ± 16.9	84.0 ± 13.1	78.0 ± 21.6	82.9 ± 14.9
VAS	48.1 ± 22.9	37.6 ± 18.1	34.2 ± 21.2	46.0 ± 31.2

^a^*P* ≤ 0.05.

^b^Outcome exceeds MCID in comparison to baseline.

Patients experienced significant improvements from baseline mHHS at 1 week (*P* = 0.03) and at 12 weeks (*P* = 0.04). In the activities of daily living (ADL) subscale of the HOS, all post-treatment measurements exceeded the MCID, while in the Sports subscale, results at 1 and 6 weeks exceeded the MCID. For the mHHS, all post-treatment measurements met the MCID threshold. For the SF-36, multiple subscales exceeded the MCID at various time points.

Five patients were able to complete GaitRite testing at baseline and at 12 weeks. The results are summarized in Table III. Neither velocity nor stride length were significantly improved at 12 weeks.

**Table III.** GaitRite analysis

	Pre-injection	Twelve weeks
Velocity (cm/s)	106.0 ± 21.7	107.5 ± 20.8
Cadence (steps/s)	106.6 ± 9.0	107.82 ± 6.2
Stride length (cm)	118.6 ± 16.7	119.4 ± 17.5
HH base support (cm)	9.4 ± 1.5	10.1 ± 2.0

## DISCUSSION

Various conclusions regarding the etiology of GTPS have been made in the past. It is now most commonly appreciated as a syndrome encompassing multiple pathologic entities. Symptoms were, at one point, attributed to ‘bursitis’ in the clinical setting, but in an early cases series of 15 patients, Karpinski *et al.* [10] did not identify any individuals with obvious objective signs of bursitis such as swelling, heat, fluctuance or crepitus. Symptoms were attributed to the pull of powerful muscles on bone, or enthesopathies, much like the etiology of supraspinatous tendinoss or tennis elbow [10]. Multiple pain generators have since been identified in literature in relation to GTPS, including multiple peritrochanteric bursae, gluteus medius and minimus, external rotators and other local structures such as the iliotibial band (ITB) [2, 11].

Of 2954 patients aged 50–79 involved in the Multicenter Osteoarthritis Study who had symptomatic knee OA or were at risk of developing symptomatic knee OA, 17.5% were noted to also have GTPS. Segal *et al.* [11] found that ITB tenderness, knee OA or pain, lower back pain and female gender were associated with GTPS in this cohort. These patients were found to have a statistically significant decrease in walking speed over 20 m and increased time to conduct multiple sit-stand exercises in comparison to patients without GTPS [11].

Imaging studies utilizing MRI, ultrasound and scintigraphy have shown the incidence of hip abductor pathology to be between 26 and 100% in patients with GTPS. Bursitis is often present along with tendon pathology, but is generally not an isolated finding in GTPS [12–17]. Others studies suggest that hip OA may be more common in patients with symptoms of GTPS. Although abnormal peri-articular findings are often present in patients with GTPS, irregular findings on imaging are also common in asymptomatic patients and reliable interpretation of imaging has proven to be difficult. This can lead to delayed diagnoses and misguided treatment in some cases [18–20].

Pathology of adjacent structures in patients with symptoms of GTPS, such as the ipsilateral femoroacetabular joint, is a common finding [21]. In their series of 15 patients with GTPS undergoing endoscopic abductor repair, all patients demonstrated labral pathology, 12 had articular cartilage damage and 9 were found to have ligamentum teres tears during the intra-articular portion of their hip arthrosocpy. Though these patients presented primarily with peritrochanteric pain, it is possible that this intra-articular pathology was the primary cause of their hip pain [4].

Local soft-tissue biopsies from patients with GTPS demonstrate more signs of pathology than matched controls, and there is an increased presence of substance P found in the bursa of these affected patients [22]. In a comparison of patients with and without hip OA, pathologic findings in the histologic study of periarticular tendon tissue were found to be more prevalent in those with hip OA [23]. In a histologic and radiographic study of murine hips, those undergoing abductor release showed greater evidence of osteoarthritic changes when compared to controls 20 weeks after injury [24].

Patients with known OA of the hip demonstrate altered gait mechanics that lead to increased lumbar lordosis and pelvic tilt in an effort to decrease hip flexion force and compensate for decreased hip motion with greater motion through other joints. These changes lead to a resultant asymmetric gait, even in early stages of OA [25]. Tendon dysfunction, altered gait and postural compensations may lead to global hip instability, which may lead to lateral hip pain [26].

Initial treatment for GTPS includes NSAIDs and PT, with a focus on stretching, flexibility, strength and gait mechanics [2]. Further treatment includes local steroid injections, with success rates reported in the literature between 50 and 100%. Conflicting evidence exists regarding the importance of the location of the injection. One study has demonstrated that whether the injection is intra- or extra-bursal at the greater trochanteric bursa does not seem to effect patient outcomes, and therefore image guidance is likely not necessary for lateral hip injections. Another has demonstrated the superiority of greater trochanteric bursal injections when compared with subgluteus medius bursal injections [27–31]. A randomized, controlled trial from the Netherlands demonstrated that that substantial subjective relief from these injections was superior to treatment with oral analgesics at 3 months post-injection (55 versus 34%; CI: 1.14–5.00), but not at 1 year post-injection (61 versus 60%; CI: 0.50–2.27) [30].

Low-energy shock-wave therapy has also been shown to be an effective alternative [32]. Multiple invasive treatment options, including debridement, tendon repair, ITB lengthening and trochanteric osteotomy have been shown to be effective in refractory cases [33–35].

The purpose of this study was to elucidate whether intra-articular steroid injections preformed in a clinic setting may help patients with GTPS who have failed to find satisfactory relief from PT, NSAIDs and lateral hip injections. This hypothesis is based on the assumption that intra-articular pathology may be the driving force for lateral hip pain in cases where traditional non-operative treatments have failed. While most outcome measures showed trends towards improvement, statistical significance was only reached with the modified Harris Hip Score at 1 and 12 weeks. GaitRite analysis showed no improvement in ambulatory performance. The MCID was met on multiple outcome studies at numerous time points, but the interpretation of this should be used with caution.

All subjects in this study were female. As previously stated, females are more prone to GTPS. The absence of male subjects, however, may make the results of this study poorly applicable to the male population.

Interpretation of patient reported outcome studies have garnered much interest in recent literature. One result is the MCID. It is important to note that the value of the MCID can be variable depending on the subjects studied and the type of intervention. Values used in this study were used in previously published studies looking at similar interventions and outcomes, but may not be exactly applicable to this group.

Limitations of this study include the lack of control group for comparison. A small study size with variable follow-up through the course of the study also makes reaching statistically significant conclusions difficult and leave the study underpowered. In addition to being underpowered, many patients did not follow up at 1, 6 and 12 weeks. A larger cohort with better adherence to protocol would make the results of this study more robust.

Having already failed traditional non-operative treatment modalities, these patients may have had more recalcitrant cases of GTPS, and it is difficult to predict what, if any, non-operative therapy may have benefitted these patients. Despite the failure of previous treatments with NSAIDs, concurrent treatment with Meloxicam around the time of the intra-articular hip injection certainly serves as a confounding variable.

In conclusion, this study showed statistically significant improvements in some measures and trends towards improvements in others after intra-articular injection for GTPS over the 12-week follow-up period. Small sample sizes and lack of a control group leave room for further research into this matter. It would be reasonable, based on the results of this study, to offer intra-articular injections for patients who have failed more traditional non-operative modalities after a period of at least 6 months.

## FUNDING

No external funding was secured for this research. Clinic visit, imaging, injection and image-guidance fees were considered standard of care in this practice and thus were billed to the patients and insurance carriers per standard protocol.

## CONFLICT OF INTEREST STATEMENT

None declared.
